# Ohio State Dental Society

**Published:** 1866-11

**Authors:** H. A. Smith


### Proceedings of Societies.

Pursuant to the following call, the gentlemen whose names are herein appended to the Constitution, met in Columbus and organized the *State Dental Society.*

*Call.*

The entire Dental Profession of the State of Ohio are hereby cordially invited to meet in mass convention, in the City of Columbus, at Naughton's Hall, on Tuesday and Wednesday the 26th and 27th of June, to form a State Dental Society, and to devise and adopt such other measures as tend to elevate and advance the interests of the profession.

Come up, brethren, and let us have such a meeting as our profession has not yet seen. The advantages to be derived from such an organization cannot be over estimated. Other states are organized. Shall Ohio lag behind?

(Signed by fifty-five dentists of the State.)

The meeting was called to order, and Dr. B. Strickland appointed Chairman, and H. A. Smith, Secretary, *pro-tem.*

On motion of Dr. Geo. Watt, the chairman appointed a committee of three, to report a *Constitution* and *By-Laws* for the use of the Society, viz: J. Taft, C. R. Butler and Geo. Watt.

On motion the following gentlemen were appointed a committee to report a Code of Ethics to the society, viz: Drs. Robinson, DeCamp, Keely, Taft and Watt.

At the request of Dr. Horton the call for meeting was read by the Secretary.

Drs. Blount, James, and Beauman were appointed a committee to report nominations for permanent officers of the Society.

The chairman of the committee on Constitution and By-Laws reported the following:

### CONSTITUTION.

### ARTICLE I.

*Name and Object.*

Sec. 1. This Association shall be called the *Ohio State Dental Society.*

Sec. 2. Its aim and purposes shall be mutual fellowship and recognition; the promoting of the honor, usefulness and interests of the profession; the advancement and cultivation of professional science and literature; the encouragement of a more thorough professional education, and the protection of the public from the evils of empiricism.

### ARTICLE II.

*Membership.*

Sec. 1. This Society shall be composed of active and honory members, who shall be selected by vote of a majority at any regular meeting, the *Committee on Membership* having previously reported them as eligible.

Sec. 2. Active members must be regular and honorable practitioners of Dentistry in the *State* of *Ohio.*

Sec. 3. Any one eligible to active membership shall not be proposed as an honorary member.

Sec. 4. Any one commencing the practice of dentistry after this date, shall not be eligible to active membership without a Diploma from a Dental College.

Sec. 5. Members, both active and honorary, shall be liable to expulsion for violation of the Constitution or By-laws, gross immorality, or unprofessional conduct, by a majority vote at any regular meeting, provided the committee on Ethics shall have reported in favor of such action at a previous meeting.

ARTICLE III.

*Officers.*

The officers of this society shall be a *President*, *two Vice-Presidents*, *a Corresponding Secretary*, *a Recording Secretary*, and a *Treasurer*, who shall be elected annually, by a majority ballot, at a regular meeting, and serve till their successors are elected. They shall perform the duties severally assigned to such officers by custom, by parliamentary usage, and the By-laws of this society.

ARTICLE IV.

*Standing Committees.*

Four standing committees, of three members each, shall be annually appointed by the President, subject to the approval of the society, as follows: an *Executive Committee*, a *Committee on Membership*, *on Publication*, and on *Dental Ethics.* These shall respectively perform the duties indicated by their names, and designated in the By-laws.

ARTICLE V.

*Amendments and By-Laws.*

Sec. 1. This Constitution may be altered or amended at any regular meeting by a two-thirds vote, provided, that notice in writing of the proposed change shall have been given at a previous regular meeting.

Sec. 2. By-Laws, in accordance with this Constitution may be adopted by a majority vote, at any regular meeting.

*By-Laws.*

Sec. 1. This Society shall hold each year an *annual* meeting on the last Tuesday in December, and a *semi-annual* meeting on the first Tuesday in June, at such places as the Society shall from time to time determine.

Sec, 2. When the officers of the Society think it advisable, the President may call a meeting at such time and place as they may have agreed on.

Sec. 3. Twelve active members shall be necessary to constitute a quorum at any meeting.

Sec. 4. In addition to his ordinary duties, the *Corresponding Secretary* shall notify members of the time and place of meetings, and of their appointments on committees, or their assignments to any special duties.

Sec. 5. The *Treasurer* shall report at the annual meeting.

Sec. 6. The *Committee on Membership*, on receiving the names of applicants, shall make due inquiry, or examination, and report to the Society, those whom they regard as worthy, who may then be elected members.

Sec. 7. All complaints against members for violating the Constitution or By-Laws, gross immorality, or unprofessional conduct, shall be presented in writing to the committee on Ethics, who shall proceed to furnish the accused a copy of the complaint, call both him and the accuser before them, examine and decide on the case, and report their decision to the next regular meeting of the Society.

Sec. 8. The *Executive Committee* shall publish an "order of business," previous to each regular meeting, provide a place of meeting &c.

Sec. 9. No one shall enjoy the rights of membership till he has signed the Constitution and By-laws, and paid an admission fee of three dollars.

Sec. 10. Each member shall be subject to an *annual tax* not exceeding *three dollars*.

Sec. 11. Official vacancies may be filled *ad interim* by the President.

Sec. 12. Parlimentary usage shall be regarded as a general guide in the transaction of business.

Sec. 13. Any of these *By-laws* may be suspended by a two-thirds vote at a regular meeting, and may be altered or amended by a similar vote, provided, notice in writing of such alteration shall have been given at a previous meeting.

The report of committee on Constitution was, on motion, accepted. The meeting then proceeded to adopt section by section, and it was then adopted as a whole.

The By-Laws as reported, were then taken up singly and adopted, after first filling blanks in No. 1, by providing for an annual meeting to be held the last Tuesday in December, and a semi-annual meeting on the 1st Tuesday of June. The By-Laws were then on motion adopted entire.

Adjourned.

### *Afternoon Session.*

Meeting convened at 2 o'clock. The minutes of morning session were read and approved.

Motion of Dr. J. Taft passed, " That we now proceed to the organization of the Ohio State Dental Society."

The Constitution and By-Laws were then read to the meeting.

*Resolution No.* 7.—The following resolution was adopted:

Resolved by this Convention, that any member of the dental profession, now present, who is a graduate of a Dental College or an active member of a local dental association in this state shall be eligible to membership in this Society.

Resolution No. 7 was now read, and the roll of members who had signed the Constitution called, each one announcing his qualification for membership in the society.

The Committee on Nominations appointed at the morning session (informal) were on motion continued. They then reported the following nominees for permanent officers of the Society:

*Nominations.*

| | | |
|---|---|---|
| For President, | Dr. | Geo. Watt, Xenia. |
| For 1st Vice " | " | G. W. Keely, Oxford. |
| For 2d " " | " | B. F. Robinson, Cleveland. |
| For Rec. Secretary, | " | H. A. Smith, Cincinnati. |
| For Cor. " | " | A. W. Maxwell, Gallion. |
| For Treasurer, | " | M. DeCamp, Mansfield. |

Signed,

C. H. James,

A A. Blount,

J. B. Beauman, } Committee.

The report was accepted on motion.

The Society then went into an election for officers with the following result:

*Officers.*

| | | |
|---|---|---|
| President, | Dr. | Geo. Watt. |
| 1st Vice " | " | G. W. Keely. |
| 2d Vice " | " | B. F. Robinson. |
| Rec. Secretary | " | H. A. Smith. |
| Cor. " | " | A. W. Maxwell. |
| Treasurer | " | M. DeCamp. |

The President elect was conducted to the chair, by Drs. Harroun and DeCamp. He then addressed the society briefly, thanking them for the honor of being called to preside over the first meeting of the Society, etc., etc.

The President named the following *Committee on Membership.*

*Committee on Membership.*—B. T. Spellman, C. H. James, F. H. Rehwinkle.

The President also appointed the followiug committees, viz:

*Committee on Publication.*—H. C. Howells, N. W. Williams, S. D. Tuttle.

*Committee on Ethics.*—B. Strickland, J. Taft, B. F. Robinson.

*Executive Committee.*—A. A. Blount, C. R. Butler, C. R. Taft.

On motion the committee on Publication was instructed to publish not less than two hundred copies of proceedings of this meeting, together with the Constitution and By-Laws in pamphlet form, for general distribution, and that the Treasurer be authorized to pay the expense of said publication.

The committee on Membership reported the following named gentlemen for membership, viz:

J. Williams, New Philadelphia; E. D. Lord, Belleview; J. McKinley, Wicksville; S. Tolbert, H. C. Howells, Hamilton; C. Bradley, E. Conway, Dayton; C. J. Cady, Delaware; S. P. Harrington, Newark; J. W. Wartman, John Fowler, Columbus; J. E. Robinson, Cleveland; J. W. French, Chillicothe; Morrison, Steubenville.

Your Committee on Membership report the above names for membership. They have been examined in part by your Committee. The other gentlemen are vouched for by members of the Society.

Signed,
B. T. Spellman,
F. H. Rehwinkle, } Com.
C. H. James,

The report was on motion accepted.

A motion was offered that the Society elect such only of the persons recommended by committee on Membership, as are present. *Passed*, after being *amended* by adding that we admit also those to membership who have authorized their names to be presented by a responsible person who is willing to pay initiation fee for them.

The persons named in the report were balloted for and elected members.

Adjourned to meet at 8 o'clock P. M.

*Evening Session.*

Society met at 8 o'clock. President in the chair. Minutes read and approved.

The Committee on Ethics reported progress. They were on motion, granted further time.

A Committee was appointed to obtain a *reporter*, to report discussions of the Society.

Dr. Taft, Chairman.

The remainder of the evening was taken up in discussing the subject of Dental Ethics.

A motion was passed that the bill recently before the State Senate be taken up and considered by the Society at the first hour in the session to-morrow.

Adjourned.

*Wednesday.—Morning Session.*

President called the Society to order. The minutes of yesterday evening session were read and approved.

The President appointed a committee of three, viz: Drs. Horton, Beauman and C. R. Taft to make nominations of delegates to the American Dental Association.

The Committee on Code of Dental Ethics reported. The report was, on motion, laid on the table.

The Secretary and Treasurer were, on motion authorized to purchase necessary books, for records at discretion.

The regular order of business for the morning was then taken up, viz: the consideration of Bill legalizing the practice of Dentistry in the State. The Bill was read by the Secretary.

The Committee authorized to secure a reporter, stated that they had suceeded in getting a suitable person. The report of committee was accepted.

Dr. Rhoades offered the following resolution:

*Resolved*, That each member of the Ohio State Dental Society be constituted a committee of one, to present to the people the importance of the Bill under consideration, being passed and becoming a law, for their and our mutual benefit. Dr. Horton moved to strike out the word "our." Lost. The resolution was then adopted.

The motion instructing the Publication committee to publish 200 copies of Constitution, By-Laws and Proceedings was resinded, and the following resolution adopted in its stead.

*Resolved,* That Dr. J. Taft be instructed to publish 500 copies of Proceedings of this Society and *Bill* (legal) and send to State legislators and to all members of the profession in the State, who do not take the *Register*, and that the Treasurer be authorized to pay the costs of said publication.

Adjourned to meet at 2 P. M.

*Afternoon Session.*

President in the chair. Minutes read and approved.

Dr. Taft offered the following, which was adopted.

*Resolved,* That a committee of five members be appointed to prepare, print, and distribute petitions in favor of the passage of the bill pending before the legislature, to the members of this and other societies, and that all members be requested to circulate them promptly, and energetically, and return them to the chairman for presentation to the legislature.

Dr. J. Taft's bill for sixteen dollars was approved and ordered paid.

The President announced the committee on Petitions, viz: Drs. Taft, James, Maxwell, Strickland, and Blount.

The following offered by Dr. Conway, was adopted.

*Resolved,* That *we*, the members of the Ohio State Dental Society, pledge themselves to sustain the Ohio Dental College, deeming it a duty we owe the profession, that its honor may be sustained, and that true Dental science may be advanced, and we further resolve that, if need be, we will give our money to sustain said institution.

The present aspect of the rubber question was taken up and fully discussed. By request, the reference to patent (Goodyear's) were not reported for publication.

Bill for use of Hall ($10) was accepted and ordered paid.

The following resolution was read and on motion laid on the table.

*Resolved,* That a committee of three be appointed to investigate the validity of the patent on vulcanized rubber, as used for dental purposes.

The Executive Committee reported as follows.

Your Committee would respectfully offer the following as the order of business, and subjects for discussion at the next meeting of the society.

1st. Reading of Minutes.
2nd. Report of Standing Committees.
3rd. Treasurer's Report.
4th. Election of Members.
5th. Election of Officers.
6th. Reading of Essays.
7th. Anæsthetics.
8th. Exostosis.
9th. Filling Teeth, Modes and Materials.
10th. Miscellaneous Business.

Your Committee would respectfully recommend to this Society that a committee be appointed to investigate the following subjects, Anæsthetics and Exostosis, and report on the same in writing.

A. A. Blount,
C. R. Butler, } Ex. Com.
C. R. Taft,

This report was on motion accepted, and adopted. The committee to nominate delegates to the American Dental Association reported, that this Society is entitled to eight delegates, and would name the following gentlemen as such delegates: Drs. B. T. Spellman, C. M. Kelsey, E. Conway, B. Strickland, C. S. Cady, H. C. Howells, W. P. Horton, F. H. Rehwinkle.

(Signed.) W. P. Horton, Chairman.

If any of the delegates appointed are not in attendance at the Dental Association, those who may be present, were on motion authorized to fill such vacancies.

The report of committee on Code of Ethics, was then on motion taken up and fully discussed, and afterwards adopted entire.

A motion was carried, that the Code of Ethics, as adopted, be published with the proceedings, etc., as provided for in resolution, No. 3.

On motion it was agreed that this Society adjourn this session at 5 P. M.

On motion the first By-Law was suspended. The Society then by vote agreed to hold the next annual meeting in Columbus, the 3rd Tuesday in January 1867.

By request, Dr. Harroun described his method of taking impressions in cases of cleft palate.

Adjourned at 5 o'clock.

*Evening Session.*

Society called to order at 8 o'clock. Minutes read and approved.

President Watt announced the appointments of committees as follows:

*Committee on Anœsthetics.*—H. A. Smith, C. H. James, H. Newington.

*Committee on Exostosis.*—C. R. Butler, B. Strickland, A. E. Lyman.

*Essayists.*—J. M. Rhoades, J. Williams, A. Maxwell, and H. C. Howells.

A motion was offered, that the President appoint a committee of three members, to report a *fee-bill* for use of society. Laid on the table.

Dr. Siddall read a paper entitled, "Ten years in the practice of Dentistry," which was, on motion, received, and referred to the Publication committee.

The subject of *Dental Education* was then taken up and fully discussed.

The following resolution was adopted.

*Resolved,* That no member of the Ohio State Dental Society shall take any man as a student, who is not known to be of good moral character, and possesed of a good education, or for a less term of pupilage than two years, and agrees to take a regular course of lectures in some Dental College.

On motion it was agreed that the next annual meeting continue three days.

A vote of thanks was tendered the Proprietors of Naughton Hall for courtesies to the Society. Adjourned.

H. A. SMITH, *Secretary.*

